# The membrane of Liliequist—a safe haven in the middle of the brain. A narrative review

**DOI:** 10.1007/s00701-020-04290-0

**Published:** 2020-03-19

**Authors:** V. Volovici, I. Varvari, C. M. F. Dirven, R. Dammers

**Affiliations:** 1 Department of Neurosurgery, Erasmus MC University Medical Center, Doctor Molewaterplein 40, 3015 GD Rotterdam, the Netherlands; 2 Center for Medical Decision Making, Department of Public Health, Erasmus MC University Medical Center, Doctor Molewaterplein 40, 3015 GD Rotterdam, the Netherlands; 3 Department of Adult Mental Health, Tees, Esk and Wear Valleys NHS Trust, York, UK

**Keywords:** Liliequist membrane, Skull base, Vascular surgery, Neuroanatomy, Surgical anatomy

## Abstract

**Background:**

The membrane of Liliequist is one of the best-known inner arachnoid membranes and an essential intraoperative landmark when approaching the interpeduncular cistern but also an obstacle in the growth of lesions in the sellar and parasellar regions. The limits and exact anatomical description of this membrane are still unclear, as it blends into surrounding structures and joins other arachnoid membranes.

**Methods:**

We performed a systematic narrative review by searching for articles describing the anatomy and the relationship of the membrane of Liliequist with surrounding structures in MEDLINE, Embase and Google Scholar. Included articles were cross-checked for missing references. Both preclinical and clinical studies were included, if they detailed the clinical relevance of the membrane of Liliequist.

**Results:**

Despite a common definition of the localisation of the membrane of Liliequist, important differences exist with respect to its anatomical borders. The membrane appears to be continuous with the pontomesencephalic and pontomedullary membranes, leading to an arachnoid membrane complex around the brainstem. Furthermore, Liliequist’s membrane most likely continues along the oculomotor nerve sheath in the cavernous sinus, blending into and giving rise to the carotid-oculomotor membrane.

**Conclusion:**

Further standardized anatomical studies are needed to clarify the relation of the membrane of Liliequist with surrounding structures but also the anatomy of the arachnoid membranes in general. Our study supports this endeavour by identifying the knowledge hiatuses and reviewing the current knowledge base.

## Introduction

Herophilus, the father of anatomy, first described the arachnoid membranes in the third century BC. The arachnoid takes its name from its cobweb-like appearance and the myth of Arachne [25].

Neuroanatomy evolves and becomes extremely detailed, taking off exponentially from the end of the nineteenth century and especially in the latter half of the twentieth century, with the advent of the operating microscope and the growing interest of neurosurgeons. In this period, a few remarkable anatomists described new details such as arachnoid cisterns [21] (Magendie) and the existence of a membrane, which splits the interpeduncular cistern in a superficial and deep part [13] (Retzius). This would later be coined the membrane of Liliequist, taking on the name of the radiologist who describes it as a membrane between two different cisterns, interpeduncular and chiasmatic [17].

The membrane of Liliequist became the central topic of several anatomical and histological studies in the 1990s. From its initial description, the basis remains, but the interpretation of subtle anatomical findings differs strongly. A precise knowledge of the exact intimate relations and morphology of the arachnoid membranes is of the utmost importance to neurosurgeons. As Yaşargil notes, aneurysms often become invested with the arachnoid walls of the cisterns, and tension on the membranes may be transmitted to the fundus of an aneurysm causing bleeding even when dissection is carried out at a distance [30].

This review aims to summarize the information published so far on the membrane of Liliequist and provides the clinical relevance in open surgery to “navigate the cisterns”.

## Materials and methods

An Embase, Ovid MEDLINE and Google Scholar search were carried out with the “membrane of Liliequist” as the main search query, without time limitations until December 2019. Papers were included if they detailed the anatomy of the membrane and its relationships with surrounding structures or if they discussed the anatomical details of the membrane in clinical context. A total of 1461 articles were screened of which 13 full text were included which detailed the anatomy of the membrane of Liliequist. Another 8 articles regarding the clinical importance of the membrane were included. Twenty-one anatomical books on the topic were manually searched, along with their references. Grey literature was searched on Web of Science. The papers were then sifted into an “anatomical landmark and description” category and a “clinical application” category.

References of all papers and books on the topic were backtracked, and references were cross-checked to ensure no papers were missed.

Risk of bias was not assessed as, at this point, no validated anatomical fixation and dissection methods exist in order to properly evaluate the arachnoid membranes. However, the implication of the preparation of heads for anatomical research is discussed and appraised.

## Gross anatomy and histology

### Morphology

The membrane of Liliequist is a partially trabecular, partially dense folded inner arachnoid membrane, and the most important anatomic landmark in the approach to the interpeduncular fossa, sellar and parasellar regions [19].

There is some variation in the description of the membrane and its anatomical borders (Table 1). Some of this variation inevitably arises from the expected variability during embryological development, but some of it arises from the technique used to prepare and dissect the cadaver heads. For example, the studies in which the membrane is described as 1 leaf are an early radiological one, of Bengt Liliequist himself [17], and one in which gelatine was infused in the subarachnoid space under high pressure, which likely resulted in the two leaves fusing together. The studies in which more than one leaf is described were done on formalin-fixed heads, which is known to cause some autolysis of the arachnoid membranes [16], and possibly some of the arachnoid trabeculae originating from the membrane were misinterpreted as extra leaves. Of these 13 anatomical studies, 1 was radiological in nature, 8 were done on formalin-fixed cadavers and 3 were performed on fresh cadavers. Notably, one study was performed by excising the sellar and parasellar region en bloc and cut into 5-μm slices for microscopic evaluation, giving another dimension to the connections between the membranes [33].

**Table 1:** The essential anatomical data and clinical relevance of included articles about the membrane of Liliequist

Study	Perforations	Number of leaves	Lateral insertion	Relation with oculomotor	Superior attachment	Type of study	Clinical relevance
Liliequist, 1959 [18]	Absent	1	N/A	N/A	Premamillary	Radiological	First description
Yaşargil, 1976 [32]	Perforated by Pcom	1	Mesiotemporal	Surrounds them	Premamillary	Intraoperative observations	Surrounds oculomotor nerve and is pierced by the posterior communicating artery, relevant during aneurysm surgery
Matsuno, 1988 [23]	Mesencephalic membrane	2	Attached to oculomotor nerves, not obvious if this is the lateral border	Attached to them	Retromamillary (posterior edge of mamillary bodies)	Cadaveric study (15); formalin-fixed heads	Forms a bridge of arachnoid membrane between the two oculomotor nerves. Also, retromamillary attachment relevant in 3rd ventriculocisternostomies.
Brasil, 1993 [2]	Pierced by oculomotor	1	Mesiotemporal	Surrounds them - arachnoidal cuff	Premamillary	Cadaveric study (7); gelatin infusion in the subarachnoid space	First description of the cuff surrounding the oculomotor nerve forming the oculomotor cistern
Zhang, 2000 [33]	Absent	2	Tentorium	Thin porous trabecular arachnoid cuff surrounds them	Free superoposterior border	Cadaveric study; sheet plastination (3), dissection (35)	Membrane extends to the tentorium, where it attaches, important in transtentorial approaches
Vinas, 2001 [29]	Pcom enters interpeduncular cistern after perforating membrane	1	Mesiotemporal	Surrounded by caudal oculomotor membrane which joins mesencephalic membrane to form membrane of Liliequist	Retromamillary	Cadaveric study (20); formalin-fixed heads submersed in Ringer’s lactate	Describes the trajectory of the posterior communicating artery which pierces the membrane before entering the interpeduncular cistern to join the Posterior Cerebral artery. This is relevant for approaches where aneurysms of the posterior communicating artery are dissected or in which posterior circulation aneurysms must be dissected from surrounding arachnoid
Lu, 2003 [21]	Pcom near inferior border of diencephalic membrane	3, diencephalic-mesencephalic leaf extra	Mesiotemporal	When posterior communicating membrane attached to Liliequist’s membrane, arachnoid cuff around nerve	Premamillary	Cadaveric study (8); formalin-fixed heads	Identifies posterior communicating artery near the border of the diencephalic membrane, relevant in 3^rd^ ventriculocisternostomies where this artery along with the posterior cerebral artery may be damaged
Froelich, 2008 [11]	Absent	2	Pia of the parahippocampal gyrus	Between two layers of mesencephalic leaf or above mesencephalic leaf, always surrounded by arachnoidal cuff until entry into cavernous sinus	Premamillary	Cadaveric study (13); formalin-fixed heads, endoscopic followed by microsurgical dissection	Explains risks of supratentorial dissection to infratentorial artery system.
Anik, 2011 [1]	Absent	2	Extends to oculomotor, gives rise to arachnoid trabeculations from then on	Variable, oculomotor usually surrounded by 1 leaf and 1 other arachnoid membrane	To the mamillary bodies themselves	Cadaveric study (24); fresh cadavers, endoscopic and microsurgical dissection	
Wang, 2011 [30]	Absent	3, hypothalamic membrane extra	Oculomotor and cerebellar tentorial incisura	Surrounded by membrane	Tuber cinereum or premamillary	Cadaveric study (15); formalin-fixed heads	Extra membrane hypothalamic membrane and adhesion to tuber cinereum which may explain hypothalamic injuries in careless dissection of piercing of the membrane.
Zhang, 2012 [33]	Absent	2	Tentorial edge and more than half of the specimens further to the uncus	Covers nerve together with another arachnoid membrane with which it unites forming the true temporal membrane which attaches mesiotemporally	Premamillary	Cadaveric study (24) and histologic study (4); Formalin-fixed heads dissected microsurgically. Histological study: sellar and suprasellar region removed en-bloc and cut into 5-μm slices	Demonstrates the extent of variability of the membrane.
Kurucz, 2013 [15]	Small perforations in the diencephalic leaf	2	Medial edge of the temporal membrane and connected to the posterior edge of the medial carotid membrane	Covers it and is connected to the lateral mesencephalic membrane under the nerve	Premamillary (75%) and retromamillary (25%)	Cadaveric study (110); fresh cadavers, endoscopic technique only, mostly through keyhole approaches	The largest study with one of the best techniques that preserves in-situ anatomy and provides a clear 3D view of the interplay between the outer arachnoid, basal arachnoid and inner arachnoid membranes.
Ciappetta, 2017 [5]	N/A	2	N/A	Surrounden by membrane	Retromamillary	Cadaveric study (10); fresh cadavers, microsurgical dissection	Just like Kurucz 2012, draws attention that the membrane originates in the basal membrane corresponding to the dorsum sellae. Trabeculae originating from the superior surface of the Liliequist membrane attach to the inferolateral surface of the optic chiasm and to the posterior and posterolateral surface of the pituitary stalk overlapping the basal arachnoid membrane.

In 54% of the included studies (7/13), the membrane of Liliequist consists of 2 leaves: a superior diencephalic and an inferior mesencephalic one [22]. Two independent studies (15%) observed that sometimes an extra leaf is present, and coined the diencephalic-mesencephalic leaf. It has a trapezoid form and is created by the union of the mesencephalic and diencephalic leaves laterally [20]**.** The possibility of a fourth leaf was coined the hypothalamic membrane. It was defined as a quadrangle with variable attachments. This was brought into question later, with new data classifying it as part of the carotid-chiasmatic wall [29].

**Fig. 1:**
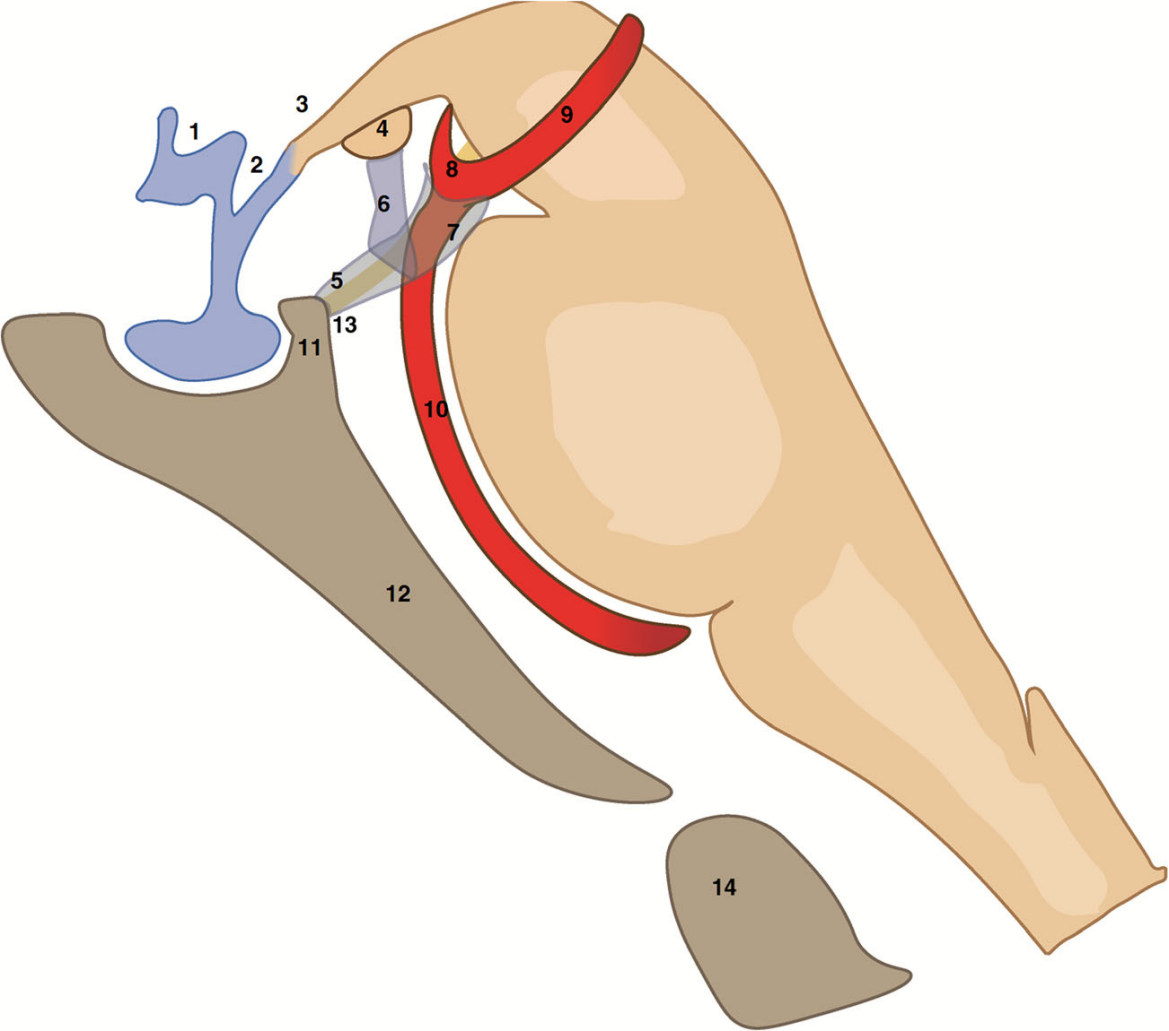
Schematic drawing of the dorsum sellae, floor of third ventricle, brain stem and interpeduncular cistern, courtesy of and designed by M. W. T. van Bilsen, MD. The supraoptic (1) and infundibular (2) recesses are depicted along with the tuber cinereum (3). The membrane of Liliequist (5, 6, 7) has a common leaf (5) which begins at the level of the dorsum sellae (11) and splits into a diencephalic leaf (6) and mesencephalic leaf (7). The diencephalic leaf (6) inserts at the level of the mamillary bodies (4), at their anterior border in 75% of cases. The mesencephalic leaf terminates just in front of the basilar apex (8) in a free border. Laterally, the membrane of Liliequist extends to the oculomotor nerve (13, in yellow). Also depicted in the figure are the left posterior cerebral artery (9), the basilar artery (10), the clivus (12) and the odontoid process (14)

The borders and attachments of the Liliequist membrane were vaguely defined in early reports. Classically, an anterior border, a posterior superior and posterior inferior border, and two lateral borders are described (Fig. 1). It originates at the level of the dorsum sellae from the basal arachnoid membrane, the part of the arachnoid which covers the skull base.

The *anterior* border of the Liliequist membrane is either situated at the dorsal clinoid processes, or dorsal to the infundibulum [2], or surrounding it, thereby establishing a hypophyseal cistern [8, 30].

The *posterior superior* border is the attachment point of the superior (diencephalic) leaf. It is described as either premamillary (8/13 studies) “at the anterior edge of mammillary bodies” [10], “pia of the posterior hypothalamus in front of the mammillary bodies” [28] or at the apex of the mamillary bodies or retromamillary (2/13 of the included studies).

The *posterior inferior* border represents the attachment point of the mesencephalic leaf. It is described to be either at the “junction of superior one third with inferior one third of basilary” [20] or at the pontomesencephalic junction or before reaching the basilar artery. Some authors mention arachnoid trabeculae that fan out from the superior border covering the posterior cerebral and posterior communicating arteries.

The *lateral* borders were delineated initially as the attachments to a separate oculomotor arachnoid sheath, and they were described as a “semilunar transverse membrane stretched obliquely between the oculomotor nerves” [6]. Others have brought this view into question and describe it as spreading beyond the oculomotor nerve to the mesial temporal surface [3, 27, 28]. Qi et al. suggest in their study that the oculomotor nerve is not a site of attachment; it merely divides the mesencephalic leaf into a medial and a lateral part [24]. The lateral attachment reaches the tentorium and forms the floor of the oculomotor cistern [1]. The authors also show that the lateral pontomesencephalic membrane is the lateral extension of the mesencephalic leaf [1]. Further research highlights that the lateral and medial pontomedullary membranes previously defined in the literature [10] are in fact continuations of the lateral and medial pontomesencephalic membranes, which in turn are the terminal parts of the mesencephalic leaf of the membrane of Liliequist. This creates a continuum of the membrane of Liliequist – pontomesencephalic – pontomedullary membranes which covers the entire anterolateral part of the brainstem.

The issue of the free borders (and thus of spaces where cisterns communicate with one another) of the membrane of Liliequist has also been the topic of several studies. The most recent view is that the membrane has a main free superior posterior border, formed by folds of fibrous bundles. Previous articles placed this free border either between the inferolateral border of the optic tracts and the uncus [2], or lateral to the oculomotor nerve [6], or in front of the basilar artery.

**Fig. 2:**
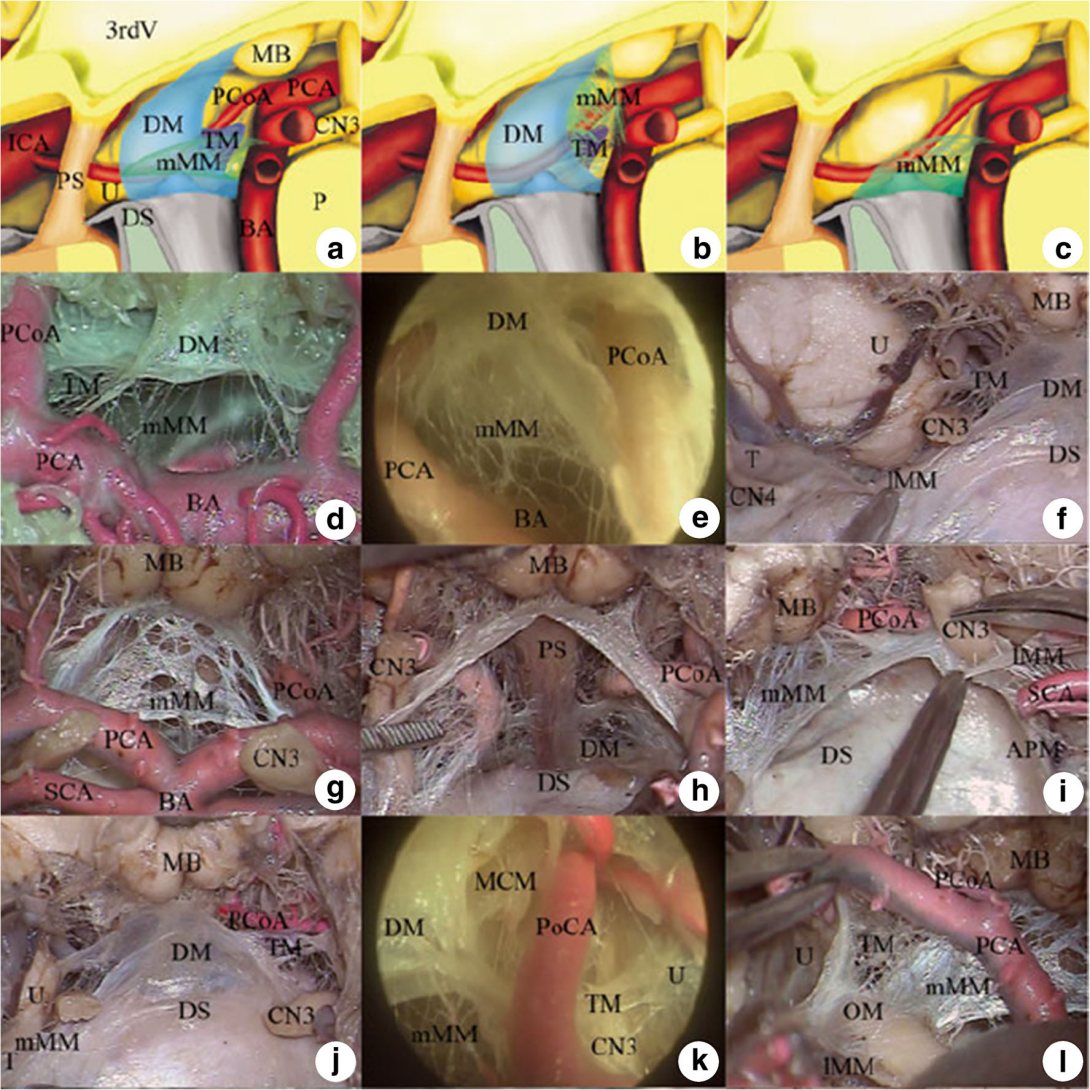
Configuration and leafs of Liliequist’s membrane in anatomical dissection. **a–c** Schematic drawing of possible configurations of the membrane as defined by Zhang et al. 2012: type I (a and b) and type II (c) of Liliequist’s membrane. **d–i** Anatomical dissection and endoscopic view of the type I Liliequist’s membrane with the oblique *Y*-shaped (d–f) or inverted oblique *L*-shaped (g–i) configuration. Note that the diencephalic membrane and/or medial mesencephalic membrane are continuous with the lateral mesencephalic membrane below the oculomotor nerve in both configurations (f and i). **j–l** Anatomical dissection and endoscopic view of the temporal membrane. Note that the temporal membrane arises directly from the lateral border of the diencephalic membrane. 3rdV third ventricle, APM anterior pontine membrane, B Basilar artery, CN3 oculomotor nerve, CN4 trochlear nerve, DM diencephalic membrane, DS dorsum sellae, ICA internal carotid artery, lMM lateral mesencephalic membrane, MB mamillary body, MCM medial carotid membrane, mMM medial mesencephalic membrane, OC optic chiasm, OM oculomotor membrane, P pons, PCA posterior cerebral artery, PCoA posterior communicating artery, PS pituitary stalk, SCA superior cerebellar artery, T tentorium cerebelli, TM temporal membrane, U uncus (Reprinted by permission from Springer Nature Customer Service Centre GmbH: [Springer Nature][Childs Nervous System][Zhang et al. Anatomical and histological study of Liliequist’s membrane with emphasis on its nature and lateral attachments][Copyright 2012])

**Fig. 3:**
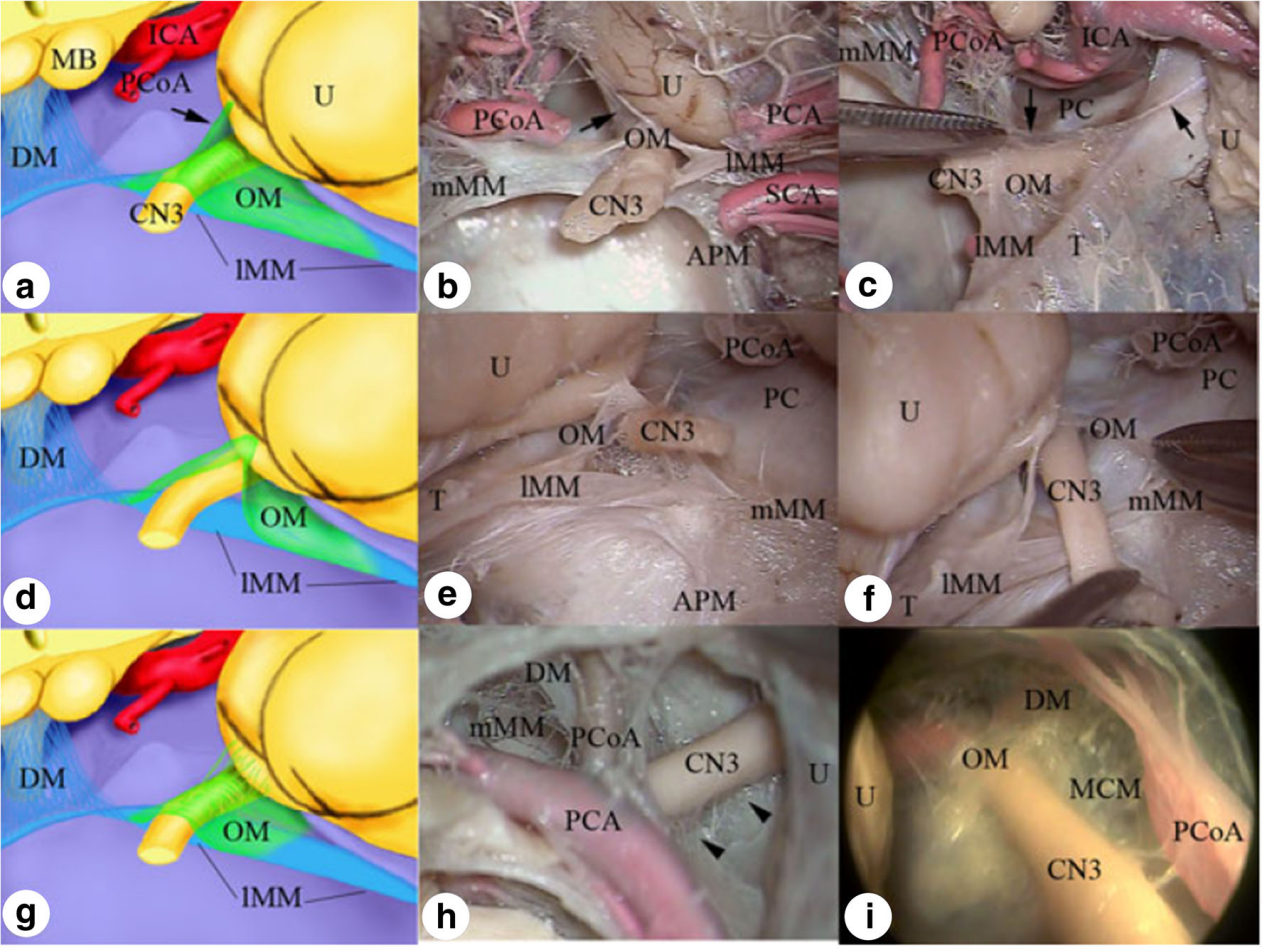
Schematic drawing and anatomical dissections showing different morphological configurations of the oculomotor membrane: the inverted Y-shaped **a**–**c**, inverted V-shaped **d**–**f** and inverted U-shaped **g**–**i**. APM anterior pontine membrane, CN3 oculomotor nerve, DM diencephalic membrane, ICA internal carotid artery, lMM lateral mesencephalic membrane, MB mamillary body, MCM medial carotid membrane, mMM medial mesencephalic membrane, OM oculomotor membrane, PCA posterior cerebral artery, PCoA posterior communicating artery, SCA superior cerebellar artery, T tentorium cerebelli, U uncus (Reprinted by permission from Springer Nature Customer Service Centre GmbH: [Springer Nature][Childs Nervous System][Zhang et al. Anatomical and histological study of Liliequist’s membrane with emphasis on its nature and lateral attachments][Copyright 2012])

Furthermore, Lu et al. describe a free superior border of the diencephalic leaf between the inferolateral border of the optic tract and the mesial surface of the temporal lobe, allowing for easy communication between the posterior communicating and carotid cisterns border [19, 20]. The posterior borders of the newly-described diencephalic-mesencephalic leaves were also seen as free border, and thus, the interpeduncular cistern could easily communicate with the crural and ambient cisterns. The mesencephalic leaf was found to have no free border [18–20] (Figs. 2, 3).

One of the largest studies and the most underrepresented in the literature is the one by Kurucz et al. [14]. Since the authors used minimally invasive craniotomies performed on 110 fresh cadaver heads and used endoscopes to explore the arachnoid spaces, this may be the most anatomically valid study. The use of fresh cadaver heads eliminated the risk of lysis of the arachnoid membrane and the minimal craniotomies and use of endoscopes ensured minimal disturbance of the in-situ anatomy. Unfortunately, because it is not PubMed indexed, it is largely unknown. Nevertheless, it provides a comprehensive view of the arachnoid membranes and cisterns, and because of the technique used, it is likely one of the most informative studies. The origin of the membrane of Liliequist from the basal arachnoid is demonstrated, its connections to the membranes lateral to the brain stem as well as its two leaves. The number of cadavers also allowed the authors enough statistical power to detect the prevalence of a premamillary and retromamillary insertion of the diencephalic leaf [14].

### Histology

Histologic studies with thinly cut microscopical slices offer even more insight into the connections of Liliequist’s membrane. In sheet plastination studies, the Liliequist’s membrane appears as a fold of the arachnoid with a double-layer structure in its core, gradually thinning towards the lateral edges, with a density very similar to the arachnoid mater, but significantly different than that of porous trabecular walls [32]. The same study calls into question whether that the arachnoid trabecular network on the anterior surface and the free border of the membrane should be considered as part of the membrane itself, since it is irregular, does not share the structure of the membrane, and varies greatly [32]. A recent study confirms these findings and labels the components of the membrane as follows: a basal part, arising from the basal part of the arachnoid membrane which appears folded and compact and an attaching part formed by many arachnoid trabeculae tied together [33].

Further histologic studies are necessary to draw conclusions regarding the embryological evolution of the arachnoid membranes and their structure as well as the arachnoid’s relationships with nearby anatomical layers. Our review was designed to address the issue of the embryological developments of the membrane of Liliequist, but the information was unfortunately not available.

### Comparison of studies and drawing a final image

The knowledge and classification of the cerebral arachnoid membranes is still developing. In fact, it is only in the more recent years that a renewed interest has surfaced to classify and describe the arachnoid membranes [14] and cisterns since Yaşargil’s description in 1984 [30].

To summarize, Liliequist’s membrane arises from the basal Membrane, courses superiorly and posteriorly, usually as a common leaf at first which then splits in two leaves: the diencephalic and mesencephalic one. The anterior border is situated at the level of the dorsum sellae and the posterior clinoid processes, where it lies in direct continuation of the Basal Membrane. The diencephalic leaf attaches posteriorly most often to the posterior portion of the tuber cinereum and anterior portion of the mamillary bodies. The lateral extension usually covers the oculomotor nerve both superiorly and inferiorly, sometimes merging with the lateral mesencephalic membrane. Together they head laterally and posteriorly towards the tentorium, the uncus and parahippocampal gyrus. The mesencephalic leaf has a free posterosuperior border in front of the basilar artery and the pons, to which it may give some arachnoid trabeculae, which are extremely variable. The posterior communicating artery and its perforators together with the oculomotor nerve may pierce the membrane. The oculomotor nerve is, during its course, surrounded by an arachnoidal cuff that is an extension of the membrane of Liliequist until its entrance into the cavernous sinus, where it gives rise to the carotid-oculomotor membrane.

## Clinical significance

The first clinical application for the membrane of Liliequist was mentioned in 1980 by Al Mefty and co-authors, citing it as the possible origin of a suprasellar arachnoid cyst in a 4-year old girl [9].

The most often cited clinical application is its role during endoscopic third ventriculocisternostomies (ETVs) [7, 11, 23]. There have been many articles which approached this topic and most of these mention the necessity to open the membrane of Liliequist during endoscopic ventriculostomy procedures [7, 11, 23]. Grand et al. describe a “cookie cutter” technique (whereby a 4.6-mm irrigating sheath was used to press and core (“cookie cut”) a section of the tuber cinereum) in the opaque floor type of the third ventricle and also insist on the importance of checking to see if there are remnants of the membrane which might block the flow of CSF [11]. In case the posterior cerebral artery P1 segment (pre-communicating) is present at the level of the area to be perforated in ETV procedures [7], the cookie cut technique is also a way to avoid injury to the P1 segment. The anatomical studies should also be taken into consideration here, as the lateral pontomesencephalic membrane slides between the posterior cerebral artery and the superior cerebellar artery [26, 32], and thus, the P1 segment can be pulled up together with the entire membrane of Liliequist. Some authors postulate that it is essential to open the membrane of Liliequist when its attachment is premamillary, but that it is not necessary to open it when the attachment is retromamillary, as the third ventricle is already open in the interpeduncular cistern. This view is not in line, however, with the description of the free borders as present in our review, and caution should be taken when applying it in surgery. Kurucz et al. draw attention to what they coin “the clival line”, a white-grey thickening on the outer arachnoid that marks the basal attachment of the brain stem arachnoid membranes [15]. Performing an ETV ventral to the clival line will lead to a subarachnoid-subdural connection and not the intended opening of the prepontine cistern, which occurs when perforating dorsally to the clival line [15].

With respect to these observations, the best way to ensure a large opening of the prepontine cistern is to open both leaves of the membrane in every procedure and to check if there are no lateral extensions along the pontomesencephalic membrane that might block the flow of CSF.

Aneurysms located anterior to the membrane of Liliequist are best approached via a pterional approach, whereas for those located behind the membrane, an orbitozygomatic or pretemporal transcavernous approach may better achieve this because the risk of injury to the vein of Labbe or temporal speech centres may be less. Tumours located anterior to the membrane of Liliequist are commonly approached by a transsphenoidal or subfrontal route [27]. In extended transsphenoidal endoscopic approaches for skull base lesions growing towards the suprasellar area, for instance, craniopharyngiomas, suprasellar cysts, pituitary adenomas with suprasellar extension or even some tuberculum sellae meningiomas, the identification of the basal arachnoid membrane and the membrane of Liliequist are essential landmarks. These act as barriers, protecting the chiasm, the superior hypophyseal arteries, which run superiorly together with the infundibulum and the perforating arteries ascending with the chiasm, tuber cinereum and mamillary bodies [29].

One essential observation has been made regarding the anatomical relations between the membrane of Liliequist and the hypothalamus: the diencephalic leaf is sometimes attached directly to the posterior surface of the infundibulum and the pituitary stalk [19], or joined to these structures and sometimes even to the tuber cinereum by dense arachnoid plexus bundles (sometimes interpreted as an extra leaf, as discussed above). The perforating arteries of the hypothalamus are also joined to the diencephalic leaf by a thick bundle of arachnoid fibres, which has essential implications for surgery involving the membrane of Liliequist due to the risk of injury to these fine vessels [19].

The membrane of Liliequist is a very important landmark in the pretemporal transcavernous approach for lesions in or around the interpeduncular fossa and an “anatomical haven” of important structures, especially basilar apex aneurysms. Yasargil [31] describes two membranes which are identified in the carotid-optic nerve–optic tract triangle, belonging to the membrane of Liliequist. [12, 20, 28].

If we summarize all the data so far, the sheath of the membrane of Liliequist along the oculomotor nerve joins the carotid arachnoid membrane and the carotid-oculomotor membrane, which used to be called the proximal ring in the past, bordering the proximal end of the clinoidal carotid.

Recently, even for trauma patients, the membrane of Liliequist has gained attention with regard to the practice of cisternostomy as advocated by Cherian and colleagues. They stipulate that opening the cisterns releases the trapped CSF and leads to better control of the intracranial pressure [4].

For both open and endoscopic approaches, the membrane of Liliequist is an important landmark, that not only guides the surgeon but is a safe haven, protecting the posterior communicating artery perforators, the oculomotor nerve and the entrance to the interpeduncular fossa, basilar apex and basilar perforators [5].

## Conclusion

The membrane of Liliequist is a very important anatomical structure in neurosurgery. There is no complete consensus about the detailed aspects of its anatomy, which underlines its complexity, and by extent the complexity of the entire arachnoid membranes-subarachnoid cisterns system. The arachnoidal cisterns around the membrane of Liliequist create a gateway for surgical procedures and form a pathway for the optimal CSF flow. The membrane itself functions as an important landmark during microsurgical and endoscopical procedures over the skull base and under the brain. Despite having been researched for more than a century, its nature, functions and secrets are still not fully understood.
